# A comparison between video otoscopy and standard tympanometry findings in adults living with human immunodeficiency virus (HIV) in South Africa

**DOI:** 10.4102/sajcd.v65i1.591

**Published:** 2018-07-16

**Authors:** Ben Sebothoma, Katijah Khoza-Shangase

**Affiliations:** 1 Department of Speech Pathology and Audiology, School of Human and Community Development, University of the Witwatersrand, South Africa

## Abstract

**Background:**

Literature suggests that there is a correlation between video otoscopy and standard tympanometry findings. However, there is limited evidence on whether these two measures are comparable in the identification of middle ear pathologies in adults living with human immunodeficiency virus (HIV).

**Objective:**

This study aimed to determine the correlation between video otoscopy and standard tympanometry with 226 Hz probe tone in the identification of middle ear pathologies in adults living with HIV in Limpopo, South Africa.

**Method:**

A prospective, non-experimental, comparative design was employed on HIV-positive adults aged 18 years and older. All participants underwent basic audiological assessment including case history interviews, video otoscopy, tympanometry with a 226 Hz probe tone and pure tone audiometry. Two ear, nose and throat (ENT) specialists independently analysed video otoscopic images and provided their reports to the researcher, and these were compared to the tympanometry results. The IBM SPSS v.24 was used for data analysis, including the use of Cohen’s kappa to determine the agreement between the two procedures. Pearson’s correlation coefficient was used to determine the strength of the correlation between tympanometry and video otoscopy.

**Results:**

A total of 87 adults (*N* = 161 ears) took part in the study. Middle ear pathology was observed in 8% (*n* = 13) of the sample when tympanometry was used, and this increased to 10.6% (*n* = 17) when video otoscopy was utilised. Kappa statistics found a good agreement (*k* = 0.7) between the diagnoses made by two ENTs. However, there was poor agreement (*k* = 0.2) between the diagnoses by video otoscopy and tympanometry. Pearson’s correlation coefficient indicated weak correlation between video otoscopy and tympanometry (*r* = 0.195).

**Conclusion:**

Findings from this study suggest that video otoscopy may be more accurate in the identification of middle ear pathologies in adults living with HIV when compared to tympanometry. These findings have training implications in the use of video otoscopy to ensure accuracy and reliability. Clinical implications of current findings include the use of both video otoscopy and tympanometry in a complementary manner for more sensitive identification of middle ear pathologies in this population. Lastly, tele-audiologic implications of the use of video otoscopy to increase access in resource-constrained contexts are raised.

## Background

The number of people living with human immunodeficiency virus (HIV) continues to increase, especially in sub-Saharan African countries (Joint United Nations Programme on HIV and/or Acquired immunodeficiency syndrome (AIDS) [UNAIDS], 2016). In South Africa, the number of people living with HIV in the general population has almost doubled from 10.6% – 19.2% in the past few years (UNAIDS, 2016). This increase occurs despite the concerted efforts to prevent the spread of HIV through various methods (Slutkin et al., 2006; Williams et al., 2006). Evidence exists that indicates that HIV increases the risk of middle ear pathologies by causing the CD4 cell count to decline in the body and thus incapacitating the immune system (Vajpayee, Negi, & Kurapati, 2013), allowing viruses and bacteria to enter the middle ear system, and causing middle ear pathologies and transient conductive hearing loss (CHL) (Chandrasekar et al., 2000; Khoza & Ross, 2002; Khoza-Shangase, 2011; Obasineke, Amdi, Ibekwe, Ezeanolue, & Ogisi, 2014; Van der Westhuizen, Swanepoel, Heinze, & Hofmeyer, 2013). Given this established link between HIV and middle ear pathologies, early identification and management of these pathologies is necessary.

Middle ear pathologies often manifest themselves on the tympanic membrane (Jaisinghani, Paparella, Schachern, & Le, 1999). The tympanic membrane undergoes colour, structural and mobility changes as a result of middle ear pathologies. These changes are also reported in adults living with HIV (Van der Westhuizen et al., 2013), regardless of whether they are on treatment or not (Fokouo et al., 2015). Some studies have reported that middle ear pathologies persist in this population despite the efficacy of antiretroviral therapy (Fokouo et al., 2015; Morsheimer, Dramowski, Rabbie, & Cotton, 2014; Tshifularo, Govender, & Monama, 2013). This occurrence of middle ear pathologies in this population highlights the importance of using sensitive measures during assessments in order to accurately identify and manage them. Studies have shown that any measure that is limited in terms of detecting tympanic membrane colour, structural and mobility changes may leave middle ear pathologies undetected (Kaf, 2011). Although transient CHL is the immediate consequence of middle ear pathologies, if left untreated, further complications may occur, and these need to be prevented through early identification.

Studies have shown that prolonged middle ear pathologies may cause permanent hearing loss and life-threatening conditions such as meningitis (Kolo, Salisu, Yaro, & Nwaorgu, 2012; Sharma, Jaiswal, Banerjee, & Garg, 2015). Hearing loss alone can affect individuals’ ability to communicate with others, especially in the presence of background noise. Furthermore, hearing loss can affect their psychosocial well-being, as well as their overall quality of life (Anderson, Parkerbery-Clark, White-Schwoch, Drehobl, & Kraus, 2013; Barker, Leighton, & Ferguson, 2017; Glyde, Cameron, Dillon, Hickson, & Seeto, 2013; Phanguphangu, 2017; Pittman, Vincent, & Carter, 2009). The presence of these conditions can complicate an already challenged quality of life in people living with HIV (Khoza-Shangase, 2017; Mutabazi-Mwesigire, Seeley, Martin, & Katamba, 2014). Several studies have indicated that people living with HIV face stigma and discrimination (Myezwa, Stewart, Musenge, & Nesara, 2009; Opara, Soni, Arinze, & Chiazor, 2013), which may affect their employment opportunities (Sprague, Simon, & Sprague, 2011). Therefore, accurately identifying middle ear pathologies may improve treatment and reduce the long-term impacts associated with these pathologies

The World Health Organization (WHO) emphasises the prevention of the impact of middle ear pathologies through early and accurate identification of these pathologies (WHO, 2011). Tympanometry with a single probe tone such as the 226 Hz remains the gold standard to evaluate middle ear function (British Society of Audiology [BSA], 2013). Testing typically involves the use of a soft nub placed into the external auditory meatus (EAM) and bouncing sound off the tympanic membrane under various amounts of pressure. The test provides immediate graphical representation of the results that indicate the presence or absence of middle ear pathologies (Martin & Clark, 2015). Several studies have shown that tympanometry with 226 Hz has a sensitivity ranging from 80% – 100% and specificity ranging from 70% – 100% (Anwar, Khan, Rehman, Javaid, & Shahabi, 2016; Harris, Hutchinson, & Moravec, 2005; Ozturk, Gode, Ogut, Bigen, & Kirazli, 2011). Although tympanometry is the gold standard for evaluating middle ear pathologies and has high sensitivity and specificity, it has been reported to be insufficient in accurately identifying all middle ear pathologies (Hunter et al., 2017; Kaf, 2011; Shahnaz & Polka, 1997), hence the importance of investigating other measures such as video otoscopy.

Video otoscopy holds promise as an alternative measure of middle ear function. Video otoscopy is based on the notion that middle ear pathologies can be diagnosed by analysing the colour and structural changes of the tympanic membrane (Jaisinghani et al., 1999). Myburgh, Van Zijl, Swanepoel, Helström, & Laurent (2016) also demonstrated that middle ear pathologies can be diagnosed automatically using tympanic membrane images. Ting, Huang and Tzeng (2016) indicated that video otoscopy produces clearer images of the tympanic membrane, which are crucial for the analysis and diagnosis of middle ear pathologies. The measure is easy and quick to use and can effectively be operated by non-health professionals with sufficient training (Biagio, Swanepoel, Adeyemo, Hall, & Vinck, 2013). Studies have shown that middle ear pathologies can be identified through the use of video otoscopy (Biagio, Swanepoel, Laurent, & Lundberg, 2014). A recent study by Ting et al. (2016) revealed a high correlation between the diagnoses made through video otoscopy and tympanograms.

Give the high prevalence of HIV (UNAIDS, 2016) with its consequent impact on middle ear function, investigations into simple, accessible, sensitive, accurate and affordable measures to accurately identify middle ear pathologies in this population are required. Early identification often leads to early intervention, preventing the consequent cost implications on medical, surgical and rehabilitation interventions for chronic ear disease. Although video otoscopic examination seems to correlate with the standard 226 Hz tone (Ting et al., 2016), there is currently limited information comparing video otoscopic findings with tympanometry in identifying middle ear pathologies in adults living with HIV, hence the importance of this study within the South African context where HIV and/or AIDS remains a significant public health challenge.

## Methods

### Aim

The aim of this study was to describe middle ear function in a group of adults living with HIV and compare the findings obtained from video otoscopy with those from standard tympanometry.

### Research design

A prospective, non-experimental comparative design was employed. A non-experimental design was used because all variables were included and described without manipulation (Irwin, Pannbacker, & Lass, 2014), with findings obtained from two measures (video otoscopy and standard tympanometry) compared to each other (Leedy & Ormrod, 2013).

### Research context

The study was conducted at Ndlovu Medical Centre (NMC) in Elias Motsoaledi Local Municipality (EMLM), Sekhukhune district, Limpopo province, South Africa. The EMLM comprises a number of rural villages with an estimated population of 249 363. The prevalence of HIV in Sekhukhune district is reported to be 17.1% (South African National Aids Council [SANAC], 2017). Because of this high prevalence, the NMC has set up an extensive HIV-oriented research consortium with Wits Reproductive Health and HIV Institute (University of Witwatersrand, Johannesburg) and the Department of Infectious Diseases, Immunology, Clinical Epidemiology and Public Health (Utrecht University, the Netherlands) (Vos et al., 2017).

### Participants’ selection criteria

Participants were selected through non-probability purposive sampling. Only participants who were diagnosed with HIV and currently taking antiretroviral treatment at NMC were invited to participate.

The inclusion that criteria were the participant must be 18 years and older, diagnosed with HIV and residing in EMLM. The exclusion criterion was individuals with discharging ears.

### Description of participants

A total of 87 adults diagnosed with HIV were recruited from NMC. All participants were attending NMC as part of their routine medical check-up and for collection of their medications. Participants were recruited using word of mouth and posters placed on the walls within the NMC.

### Data collection

A self-developed abstraction sheet was used to collect pertinent case history data from all participants. Video otoscopy examination using Otocam of the Amtronix connected to an Acer laptop was used to capture the tympanic membrane images. Tympanometry with 226 Hz probe tone Titan, 3.3 version of the Interacoustics was used to evaluate the mobility of the tympanic membrane. Pure tone thresholds were obtained using the AD629 diagnostic audiometer of the Interacoustics, with Sennheiser HA200 supra-aural headphones and Radio Ear B-71 bone conductor. A sound-treated audiometric two-room suite was used to conduct all the tests (Martin & Clark, 2015).

Participants were interviewed to obtain pertinent information about symptoms of middle ear pathologies, as well as all other significant medical history. The researcher conducted a basic audiological test battery, tympanometry, as well as video otoscopy on all participants. Video otoscopic images were saved under a special folder on the computer using codes. These images were sent to two ENT specialists using asynchronous telemedicine (save and forward) (Biagio et al., 2013). The ENT specialists were requested to independently analyse the video otoscopic images and indicate the presence or absence of middle ear pathology. The two ENT specialists had a total of over 20 years of clinical experience and were registered with the Health Professional Council of South Africa (HPCSA).

### Reliability and validity

Test–retest reliability was achieved by conducting a pilot study prior to the main study (Satake, 2015). This pilot study was conducted on five adults who met the same inclusion criteria of the main study. No changes were required in the design and tools of the study following pilot study findings. All measures were calibrated according to American National Standards Institute (ANSI). Daily biological calibration was also conducted before testing participants on each day (Wilber & Burkard, 2015).

### Data analysis

Raw data were first captured on a Microsoft Excel spreadsheet. The IBM Statistical Package for Social Sciences (SPSS) version 24 was used to analyse the data. Descriptive statistics including frequency calculations and percentages were used to summarise the results and describe the characteristics of the sample (Irwin et al., 2014). Kappa statistics were used to determine the agreement between the diagnoses of the two ENT specialists, and between the ENT diagnosis and the tympanometry (Viera & Garreth, 2005). A Pearson’s correlation coefficient was used to determine the strength of the correlation between video otoscopy and tympanometry data. Correlation coefficients are expressed as values between +1 and -1, with +1 indicating a perfect positive correlation, while 0 indicates no correlation. Values between 0 and 0.3 (0 and -0.3) indicate a weak positive (negative) correlation, while values between 0.3 and 0.7 (-0.3 and -0.7) indicate a moderate positive (negative) correlation. Values between 0.7 and 1 (-0.7 and -1) indicate a strong positive (negative) correlation (Mukaka, 2012).

## Ethical consideration

The authors assert that all procedures contributing to this work comply with the ethical standard of the relevant national and institutional guidelines on human experimentation (The University of the Witwatersrand Medical Ethics committee) and with the Declaration of Helsinki, 2013. The study adhered to the ethical principles outlined in the Declaration of Helsinki (World Medical Association Declaration of Helsinki, 2013). Ethics approval was obtained from the Human Research Ethics Committee (HREC) (medical) of the University of the Witwatersrand, Johannesburg (M160663).

## Results

Participant demographics includes age, gender, ethnicity and treatment information: type of regimen and duration of treatment.

### Description of the participants

The study was comprised of 99 (198 ears) adults diagnosed with HIV. The sample, as depicted in Table 1, included 28% males and 72% females, with a mean age of 46 years (25–72 years); 99% of the participants were taking antiretroviral therapy as part of their treatment regimen, with only 1% treatment naïve. The names of the treatments per specific patient were not documented; however, the ARV treatment offered at the NMC is in line with the adult antiretroviral therapy guidelines (Meintjies et al., 2017). Because of poor visualisation quality of some of the video otoscopic images, analysis and diagnosis could not be made in 25 ears (13%), and therefore, these ears were excluded from the study. A total of 173 ears were, therefore, included for further analysis.

**TABLE 1:** Summary of results.

Factors	Variables	%	*n*
Gender	Males	28	24
	Females	72	63
Tympanometry	Type Ad	70	9
	Type As	15	2
	Type B	15	2
	Type C	0	0
Visualisation quality	Good quality	87	173
	Poor quality	13	25
Middle ear pathology	Tympanometry	8	13
	Video otoscopy	11	17

Note: The range: 25–72 years, with a mean age of 46 years.

### Middle ear assessment findings

#### Occurrence of middle ear pathologies

The occurrences of middle ear pathologies in this study were based on the agreement of the diagnoses of the total sample (161 ears). Video otoscopy indicated an occurrence of middle ear pathologies in 11.5% of the sample, with the rest presenting with normal middle ear function. However, tympanometry revealed an occurrence of middle ear pathologies in 8% of the sample, as depicted in Table 1. Of the 8% with middle ear pathologies through tympanometry, type Ad tympanogram was the majority (70%).

The types of middle ear pathologies found included otitis media with effusion (*n* = 5), retracted tympanic membrane (*n* = 4), tympanic membrane perforations (*n* = 3), chronic suppurative otitis media (*n* = 2), tympanosclerosis (*n* = 1), tympanic membrane scarring (*n* = 1) and otitis externa (*n* = 1).

### Inferential statistics

The video otoscopic findings and the standard tympanometry findings were compared. Of the 173 ears, the two ENT specialists did not agree on the diagnoses of 12 ears (16%). Cohen’s kappa (*k* = 0.7) revealed a substantial agreement between the diagnoses made by the two ENT specialists using video otoscopic images. The 12 ears that ENTs could not agree on their diagnoses were not included when comparing the video otoscopy and tympanometry. Therefore, a total of 161 ears were used to compare the two measures.

When comparing the diagnoses made through the video otoscopy and tympanometry, Cohen’s kappa (*k* = 0.2) indicated poor agreement between the two measures. Of the middle ear pathologies identified by both measures, the two measures only agreed on four diagnoses. A Pearson’s correlation analysis revealed a weak correlation of 0.19 (*n* = 161; *p* = 0.13) between the two measures.

## Discussion

The aim of this study was to describe middle ear function in a group of adults living with HIV and also compare their video otoscopic findings with standard tympanometry findings. This study found that middle ear pathology was present in this sample. The occurrence rate increased from 8% of the sample when tympanometry was used to 11% when video otoscopy was utilised. The most commonly found middle ear pathologies were otitis media with effusion and retracted tympanic membrane. Although kappa statistics indicated good agreement between the diagnoses made by two ENTs, there was poor agreement between the diagnoses by video otoscopy and tympanometry. Pearson’s correlation coefficient indicated weak correlation between video otoscopy and tympanometry. Current findings add to the existing scientific evidence on middle ear pathologies in adults living with HIV. Although the sample of this study was small when compared to previous studies on adults living with HIV (Khoza-Shangase, 2018; Van der Westhuizen et al., 2013), the age and gender distribution of the sample can be considered a fair representation of the South African population living with HIV, with the majority being women living with HIV (Statistics South Africa [SSA], 2017).

The occurrence of middle ear pathologies in individuals living with HIV in this study was evident from both the video otoscopic findings and tympanometry findings. In this study, the occurrence of middle ear pathologies ranged between 8% and 11%, with video otoscopy identifying more pathologies (11%, *n* = 17) than tympanometry (8%, *n* = 13). The occurrence of middle ear pathologies in this study is consistent with published literature indicating that middle ear pathologies are common in individuals living with HIV (Chandrasekar et al., 2000; Obasineke et al., 2014; Tshifularo et al., 2013; Van der Westhuizen et al., 2013). Although the occurrence of middle ear pathologies is evident in this study, it is noted that these occurrences are slightly lower when compared with the previous studies (Tshifularo et al., 2013; Van der Westhuizen et al., 2013). The low occurrence in this study may be attributed to difference in sample sizes and methodological approaches adopted. Despite the differences in occurrence of the middle ear pathologies, current findings confirm that middle ear pathologies exist in individuals living with HIV, despite the known efficacy of antiretroviral therapy (ART) – which almost all participants in this study were on (Morsheimer et al., 2014).

When comparing the occurrence of middle ear pathologies based on the measures used, current findings indicated that the results obtained from the two ENT specialists were comparable with a kappa statistics of 0.7, indicating substantial agreement (McHugh, 2012). These findings are supported by evidence which indicates that there is good inter-tester agreement between ENTs when using video otoscopy (Biagio et al., 2013; Lundberg, Westman, Hellstrom, & Sandstrom, 2008). In this study, both ENT specialists excluded 13% of the ears from analysis because of poor visualisation quality. Good tester agreement with this measure has important implications for widespread use of video otoscopy where tester and test–retest reliability are important. This is particularly important in resource-constrained contexts such as South Africa, where non-professionals might need to be trained to take these images with analysis conducted remotely via tele-practice. Furthermore, the small percentage of poor visualisation quality images (13%) in this study is a further encouraging positive finding for video otoscopy as a measure and is consistent with results by Mbao, Eikelboom, Atlas and Gallop (2003) who found that the total number of poor quality images that could not be analysed was less than 16%. Current findings on the number of acceptable video otoscopic images for analysis were also similar to those reported by Biagio et al. (2013).

Although Ting et al. (2016) reported a high correlation between the video otoscopic findings and tympanometry with 226 Hz, current findings revealed a weak correlation between the diagnoses made using video otoscopic images and tympanometry (*r* = 0.19). Video otoscopy yielded a higher occurrence of middle ear pathologies (11%, *n* = 17) than tympanometry with 226 Hz (8%, *n* = 13). The higher occurrence obtained through video otoscopy suggests that middle ear pathologies may affect the structures and colour of the tympanic membrane before (or without) affecting its mobility (Hunter et al., 2017), aspects of middle ear function that are not always measurable by tympanometry. Several studies that have compared tympanometry with 226 Hz and other middle ear measures have also indicated that tympanometry may miss middle ear pathologies that have not yet affected the mobility of the tympanic membrane by up to 63% (Kaf, 2011; Shahnaz & Polka, 1997). This is problematic where early identification is a goal as mobility might be impacted long after the initial onset of the middle ear pathology.

Current findings suggest that tympanometry alone cannot provide accurate information about middle ear functioning in adults living with HIV, particularly if mobility disorder is not a key or initial feature of the middle ear pathology. These findings seem to suggest that audiologists and other hearing health professionals need to use measures that provide sufficient information about the characteristics of the tympanic membrane, which include structure, colour and mobility in order to achieve the goal of early identification of middle ear pathologies in this population. Although pneumatic otoscopy provides such sensitivity and specificity in terms of middle ear assessment (Ponka & Baddar, 2013), it is not a measure that is useful in a resource-constrained context as it requires specialised skills from trained physicians (Abbott, Rosenkranz, Hu, Gunasekera, & Reath, 2014). Because of the documented shortage of physicians in developing countries (Fagan & Jacobs, 2009; Mulwafu, Ensink, Kuper & Fagan, 2017), pneumatic otoscopy may not be a feasible measure for this purpose within the South African context.

Current findings have important clinical implications. Good prognosis has been documented where middle ear pathologies are identified and treated early. Consequences of untreated middle ear pathologies are significant for both patient and the State, and this has been reported by Phanguphangu (2017). It is evident from this study that the use of tympanometry alone may leave other middle ear pathologies unidentified, especially pathologies that have not yet affected the mobility of the tympanic membrane. Given that individuals living with HIV present with a number of middle ear pathologies, varying in degree and severity (Obasineke et al., 2014; Tshifularo et al., 2013), an accurate measure of middle ear function that can identify early signs of these pathologies is warranted, hence the implications of current findings on the use of video otoscopy as part of the test battery.

Although the findings of this study are significant for assessment and management of middle ear function of individuals living with HIV, the results should be interpreted with caution because of the identified methodological limitations. These limitations include the small sample size and the fact that participants were sampled from only one province in South Africa.

## Conclusion

Middle ear pathologies in individuals living with HIV exist despite the efficacy of highly active antiretroviral treatment, albeit in small numbers when compared to treatment naïve individuals. If these pathologies are not detected and treated early, they may contribute significantly to the burden of hearing loss which already exists in developing countries. Tympanometry may not always accurately identify middle ear pathologies in individuals living with HIV. The use of video otoscopy in a complementary manner with tympanometry with 226 Hz may contribute to early and accurate identification of middle ear pathologies in this population. Moreover, the use of video otoscopy can contribute significantly in the accurate identification of middle ear pathologies, with the tele-practice capabilities presenting significant possibilities for access in resource-constrained contexts. Therefore, hearing health professionals need to consider using both video otoscopy and tympanometry with 226 Hz probe tone in order to get an accurate indication of middle ear function of not only individuals living with HIV but the general population as well.
